# Sero-Prevalence of Rodent Pathogens in India

**DOI:** 10.1371/journal.pone.0131706

**Published:** 2015-07-09

**Authors:** Shrruthi Manjunath, Prachet G. Kulkarni, Krishnaveni Nagavelu, Rosa J. Samuel, Sandhya Srinivasan, Nandhini Ramasamy, Nagendra R. Hegde, Ramachandra S. Gudde

**Affiliations:** 1 Central Animal Facility, Indian Institute of Science, Bengaluru, India; 2 Ella Foundation, Genome Valley, Turkapally, Shameerpet Mandal, Hyderabad, India; Institut National de la Santé et de la Recherche Médicale (INSERM), FRANCE

## Abstract

Health monitoring is an integral part of laboratory animal quality standards. However, current or past prevalence data as well as regulatory requirements dictate the frequency, type and the expanse of health monitoring. In an effort to understand the prevalence of rodent pathogens in India, a preliminary study was carried out by sero-epidemiology. Sera samples obtained from 26 public and private animal facilities were analyzed for the presence of antibodies against minute virus of mice (MVM), ectromelia virus (ECTV), lymphocytic choriomeningitis virus (LCMV), mouse hepatitis virus (MHV), Sendai virus (SeV), and *Mycoplasma pulmonis* in mice, and SeV, rat parvo virus (RPV), Kilham’s rat virus (KRV) and sialodacryoadenitis virus (SDAV) in rats, by sandwich ELISA. It was observed that MHV was the most prevalent agent followed by *Mycoplasma pulmonis* and MVM in mice, and SDAV followed by RPV were prevalent in rats. On the other hand, none of the samples were positive for ECTV in mice, or SeV or KRV in rats. Multiple infections were common in both mice and rats. The incidence of MHV and *Mycoplasma pulmonis* was higher in facilities maintained by public organizations than in vivaria of private organizations, although the difference was not statistically different. On the other hand the prevalence of rodent pathogens was significantly higher in the northern part of India than in the South. These studies form the groundwork for detailed sero-prevalence studies which should further lay the foundations for country-specific guidelines for health monitoring of laboratory animals.

## Introduction

The suitability of an animal species for a specific experiment is dictated by the physiology of the animal [1]. Pathogens of laboratory rodents cause subclinical infections which can influence physiological and pharmacological parameters, potentially influencing the interpretation and outcome of experiments conducted on such animals [2], [3]. Several microorganisms are known to influence the function of various organ systems, affecting normal physiological values of indicator biomolecules in mice, rats and rabbits, without causing clinical signs [4]. Subclinical infections may also be exacerbated to produce overt disease by experimental procedures. Such infections may also increase variability among experimental animals, resulting in usage of more number of animals to achieve statistically significant results, further leading to misinterpretations or inconclusive results. Therefore, the use of minimum number of experimental animals that are free from unwanted microorganisms is an important pre-requisite to achieve reliable and reproducible results. Consequently, the microbiological status of both individual animals and the population as a whole, and therefore periodic and systematic health monitoring, plays a critical role in assessing the quality and the suitability of laboratory animals for experiments [5], [6]. Proper health monitoring is also important to validate the efficiency of measures undertaken to prevent the introduction of pathogens.

The identification of agents prevalent at individual or population level can be achieved by sero-epidemiology or other studies [7]. Following initiatives in the US and Japan in the 1980s, microbiological monitoring of laboratory animals was expanded to 100 mouse and rat pathogens which potentially interfere with biomedical research. In 1994, the WHO and the International Council for Laboratory Animal Science (ICLAS) published guidelines for the breeding and care of laboratory animals, and this included health monitoring [8], [9]. In Europe, frequent monitoring is recommended only for the most prevalent agents, with less frequent monitoring for the rare agents [10]. A number of suggestions for establishing health monitoring programmes have been published in the past few decades [11–13] and the importance of global standards and harmonization has also been stressed [14].

India has emerged as a significant player in the global biotech arena, with large-scale outsourcing of work by several pharmaceutical drug discovery firms. Consequently, state-of-the-art facilities established by many contract research organizations and pharmaceutical industries have opted for accreditation of their vivaria by India’s National Good Laboratory Practice Compliance Monitoring Authority as well as the Association for Assessment and Accreditation of Laboratory Animal Care International (AAALAC), USA, in order that study reports are acceptable globally. However, these have been mostly need-based and driven by regulatory requirements, and not standard practices. Of the more than 1400 animal facilities registered with the Committee for the Purpose of Control and Supervision of Experiments on Animals (CPCSEA), which regulates animal experimentation in India, very few meet international standards and many do not have health monitoring programmes due to prohibitive costs. In addition, no systematic data is available on the prevalence of laboratory rodent pathogens in India. The present study was undertaken to determine the prevalence of various rodent pathogens in laboratory animals in some parts of India where majority of the animal facilities are located.

## Materials and Methods

### Collection of Samples

Serum samples (500 μL) were sought from five mice and five rats maintained at 26 vivaria in public and private organizations located in different parts of India. These samples were either collected by a veterinarian or by the trained personnel under the supervision of a veterinarian. Majority of the animal facilities obtained Institutional Animal Ethics Committee (IAEC) approval specific to the health monitoring programme in their respective institutes/organizations, and the rest collected serum samples from the control group of animals from the on-going IAEC approved animal experiments. The sera were shipped on freezer packs and stored at -80°C until further analysis.

### Enzyme-linked Immunosorbent Assay (ELISA)

The serum samples were analyzed for the presence or absence of antibodies against for minute virus of mice (MVM), ectromelia virus (ECTV), lymphocytic choriomeningitis virus (LCMV), mouse hepatitis virus (MHV), Sendai virus (SeV), and *Mycoplasma pulmonis* in mice, and SeV, rat parvovirus (RPV), Kilham’s rat virus (KRV) and sialodacryoadenitis virus (SDAV) in rats, by sandwich ELISA [15], using commercial kits (XpressBio Life Science Products, Thurmont, MD, USA), as per instructions provided by the manufacturer. Briefly, 100 μL of 50-fold diluted test, negative control and positive control sera were pipetted into appropriate wells, covered and incubated at 37°C for 45 min. The wells were then washed thoroughly five times, filled with 100 μL per well of ready-to-use peroxidase conjugate, and incubated at 37°C for 45 min. The wells were washed again, and a 100 μL of ready-to-use ABTS [2,2'-azino-bis(3-ethylbenzothiazoline-6-sulphonic acid)] peroxidase substrate was added to each well before incubating at room temperature for 30 min. The extent of reactivity was extrapolated from the colorimetric reaction which was assessed by reading absorbance at 405 nm. The sample was considered positive if the difference in absorbance of the sample between the Positive Viral Antigen well and the Negative Control Antigen well was ≥ 0.300.

### Statistical Analysis and Generation of Graphs

The data between samples from government and private organizations as well as between those from North and South India were analysed for statistical significance by the paired *t*-test. GraphPad Prism (Version 5) was used for the preparation of graphs. EPI-Infosoftware (Centres for Disease Control and Prevention, Atlanta, USA) and digitized maps of India were used for preparing distribution of pathogens.

## Results

One hundred and thirty serum samples each from mice and rats were analyzed for six pathogens of mice and four pathogens of rats by ELISA. For the examined set of pathogens, 53.84% of the mice were found to be infected with one or more pathogens whereas the rate of infection in rats was substantially lower (20%).

In mice, the incidence of MHV was high (46.92%), and this was followed by *Mycoplasma pulmonis* (22.30%) and MVM (13.84%). On the other hand, the incidence of SeV and LCMV were very low (2.30% and 0.76%, respectively), and none of the samples were positive for ECTV (Table 1). In rats, the incidence was highest for SDAV (16.15%); 5.38% of the samples were positive for RPV, and none of the samples were positive for SeV or KRV (Table 1).

**Table 1:** Incidence of various pathogens in mice and rats.

Pathogen	Mice		Rats	
	No. positive	% incidence	No. positive	% incidence
Mouse hepatitis virus (MHV)	61/130	46.92		
*Mycoplasma pulmonis*	29/130	22.30		
Minute virus of mice (MVM)	18/130	13.84		
Sendai virus (SeV)	3/130	2.30	0/130	0.00
Lymphocytic choriomeningitis virus (LCMV)	1/130	0.76		
Ectromelia virus (ECTV)	0/130	0.00		
Sialodacryoadenitis virus (SDAV)			21/130	16.15
Rat parvovirus (RPV)			7/130	5.38
Kilham’s rat virus (KRV)			0/130	0.00

Fifty four per cent of the sera tested positive for one or the other agent, and multiple infections were common, with 14.75% of the sera being positive for three or more agents. In mice, MHV appeared to co-exist with other pathogens (55.74%) rather than singly (39.34%). On the other hand, MVM was never found singly (Table 2). Whereas all the positive samples from rats were for parvoviruses, 11.54% of the samples were positive for both RPV and SDAV, while 73.08% were positive for SDAV only.

**Table 2:** Prevalence (no. of positive samples) of multiple infections in mice and rats.

Frequency of mouse pathogens		Frequency of rat pathogens	
MHV	61	SDAV	21
*M*. *pulmonis*	29	RPV	7
MHV, *M*. *pulmonis*	26	SDAV, RPV	3
MHV, MVM	18		
MHV, MVM, *M*. *pulmonis*	09		
MHV, MVM, SeV, *M*. *pulmonis*	02		
MHV, MVM, LCMV, SeV, *M*. *pulmonis*	01		

MHV, mouse hepatitis virus; *M*. *pulmonis*, *Mycoplasma pulmonis*; MVM, minute virus of mice; SeV, Sendai virus; LCMV, lymphocytic choriomeningitis virus.

The prevalence of various mouse and rat pathogens in southern and northern parts of India was also analyzed. More number of samples was procured from South India, which include Karnataka, Tamilnadu, Andhra Pradesh, Maharashtra and Kerala States, where the majority of the animal facilities are located. The northern states include the States of Gujarat, West Bengal, Haryana, Delhi and Uttar Pradesh. It was observed that the incidence of MHV and *Mycoplasma pulmonis* in mice was more in North India (57.14% and 31.42%, respectively) compared to South India (43.15% and 18.94%, respectively), and the only positive sample for LCMV was from North India. In rats, the incidence of SDAV and RPV was higher in North India (25.71% and 11.42%, respectively) compared to South India (12.63% and 3.15%, respectively) (Table 3). Overall, the incidence of rodent pathogens in North India was significantly higher than in South India (p = 0.0047).

**Table 3:** Incidence (%) of mouse and rat pathogens in North (35 samples) and South (95 samples) India.

Pathogen	South India	North India
Mouse hepatitis virus (MHV)	43.15	57.14
*Mycoplasma pulmonis*	18.94	31.42
Minute virus of mice (MVM)	11.57	20.00
Sendai virus (SeV)	1.05	5071
Lymphocytic choriomeningitis virus (LCMV)	0.00	2.85
Sialodacryoadenitis virus (SDAV)	12.63	25.71
Rat parvo virus (RPV)	3.15	11.42

**Fig 1:**
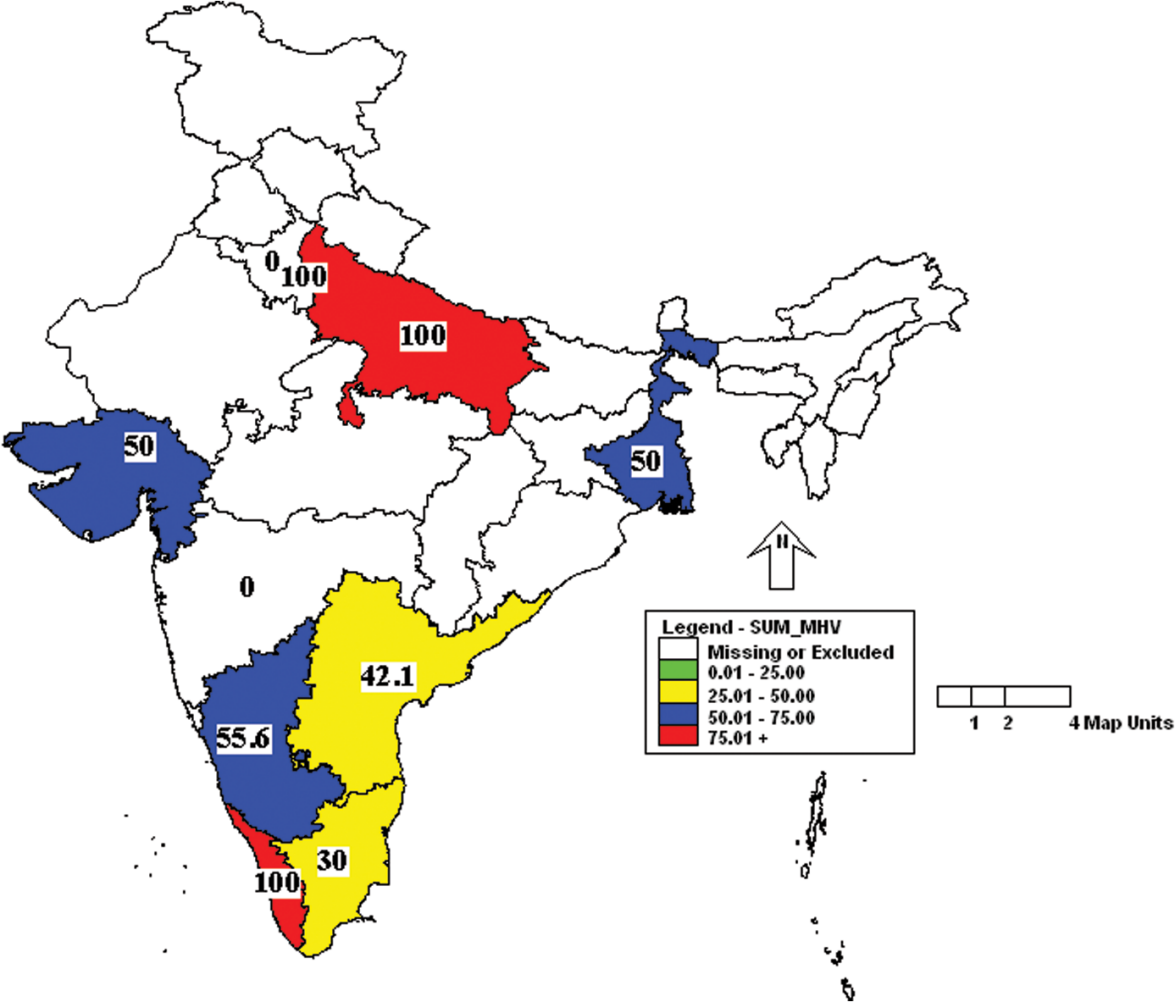
Incidence of mouse hepatitis virus (MHV) in different states of India. Yellow—25.01 to 50.00%; Blue—50.01 to 75.00%; Red—75.01 to 100.00%.

**Fig 2:**
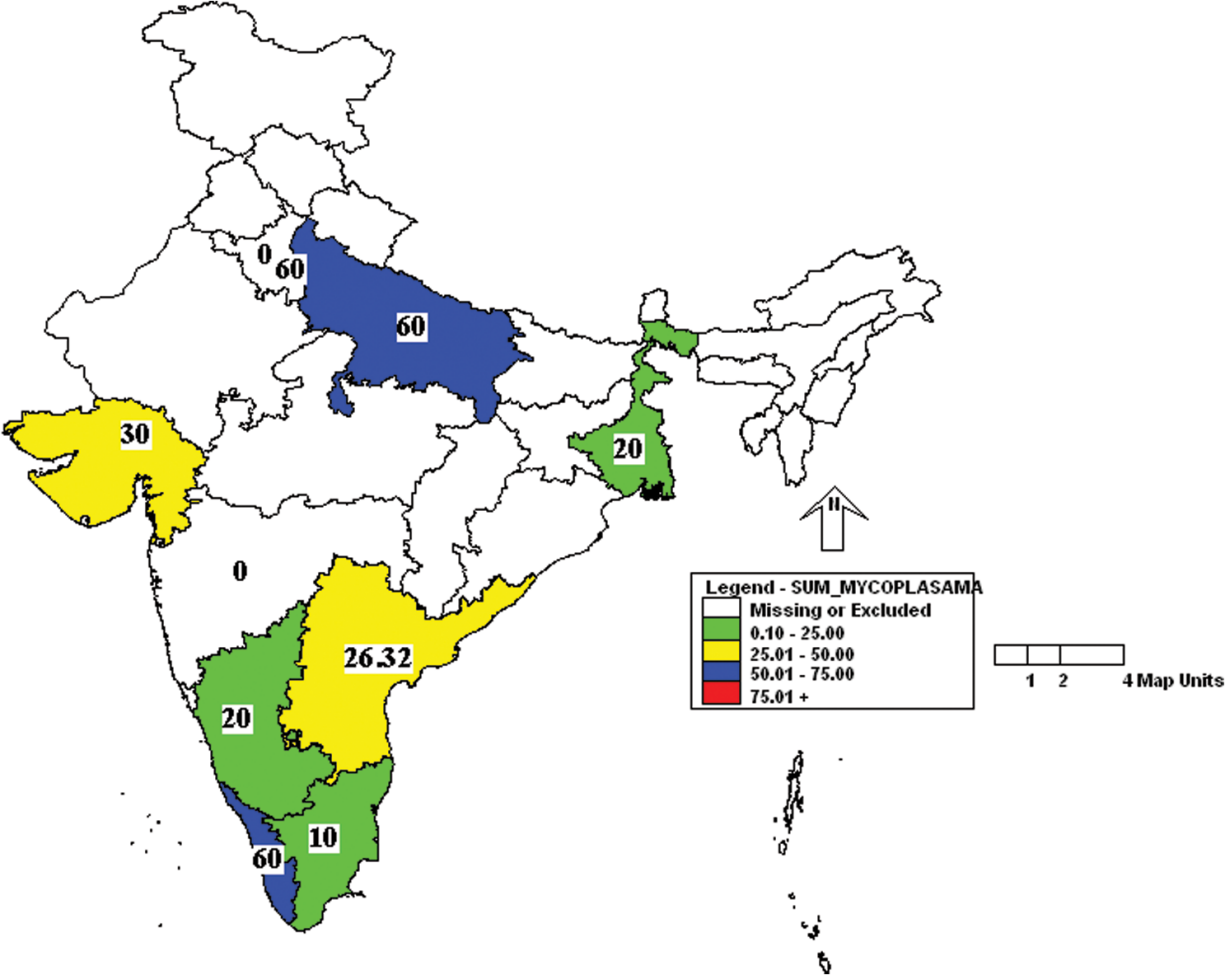
Incidence of *Mycoplasma pulmonis* in different states of India. Green—00.00 to 25.00%; Yellow—25.01 to 50.00%; Blue—50.01 to 75.00%.

**Fig 3:**
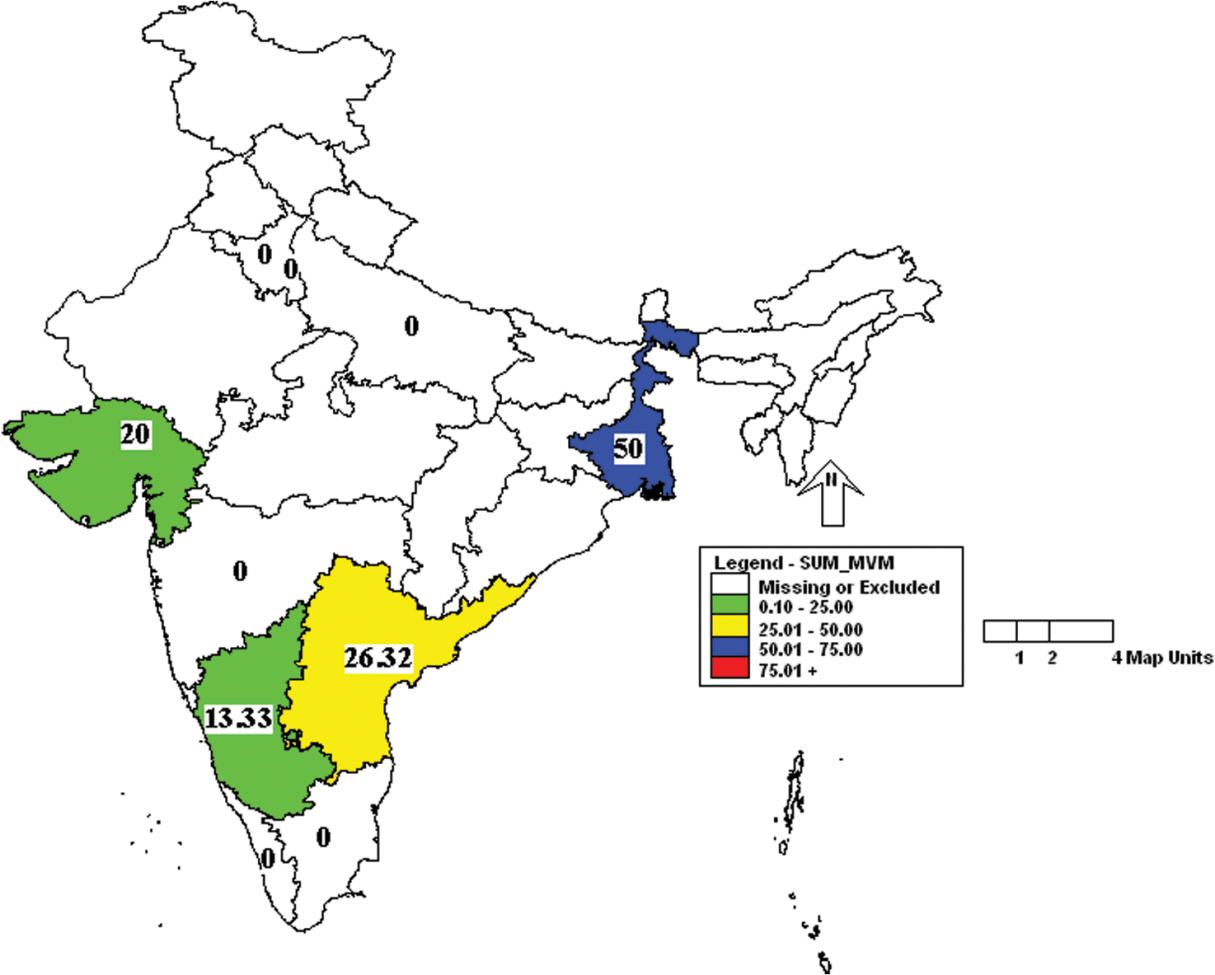
Incidence of minute virus of mice (MVM) in different states of India. Green—00.00 to 25.00%; Yellow—25.01 to 50.00%; Blue—50.01 to 75.00%.

**Fig 4:**
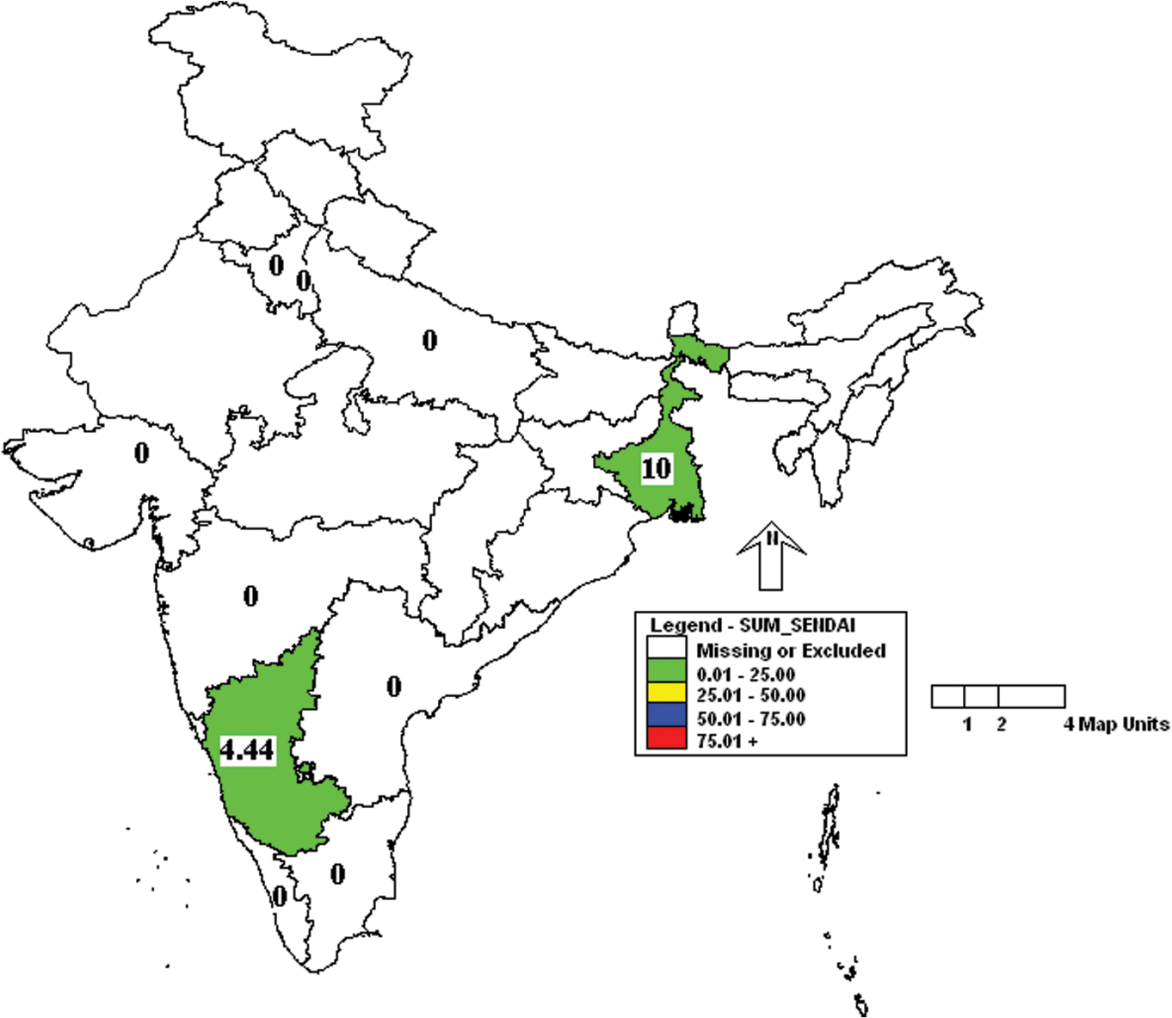
Incidence of Sendai virus (SeV) in different states of India. Green—00.00 to 25.00%.

**Fig 5:**
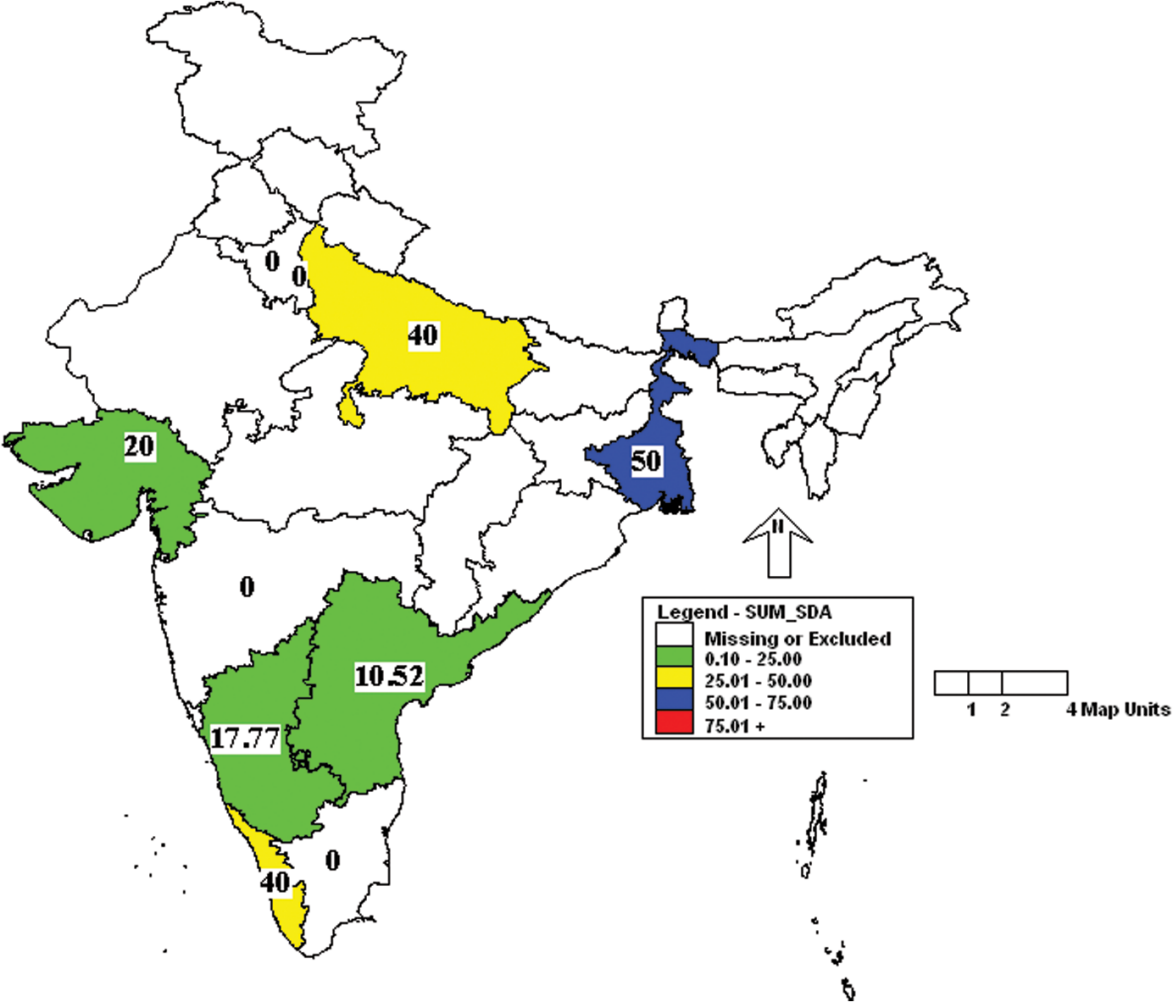
Incidence of Sialodacryoadenitis virus (SDAV) in different states of India. Green—00.00 to 25.00%; Yellow—25.01 to 50.00%; Blue—50.01 to 75.00%.

**Fig 6:**
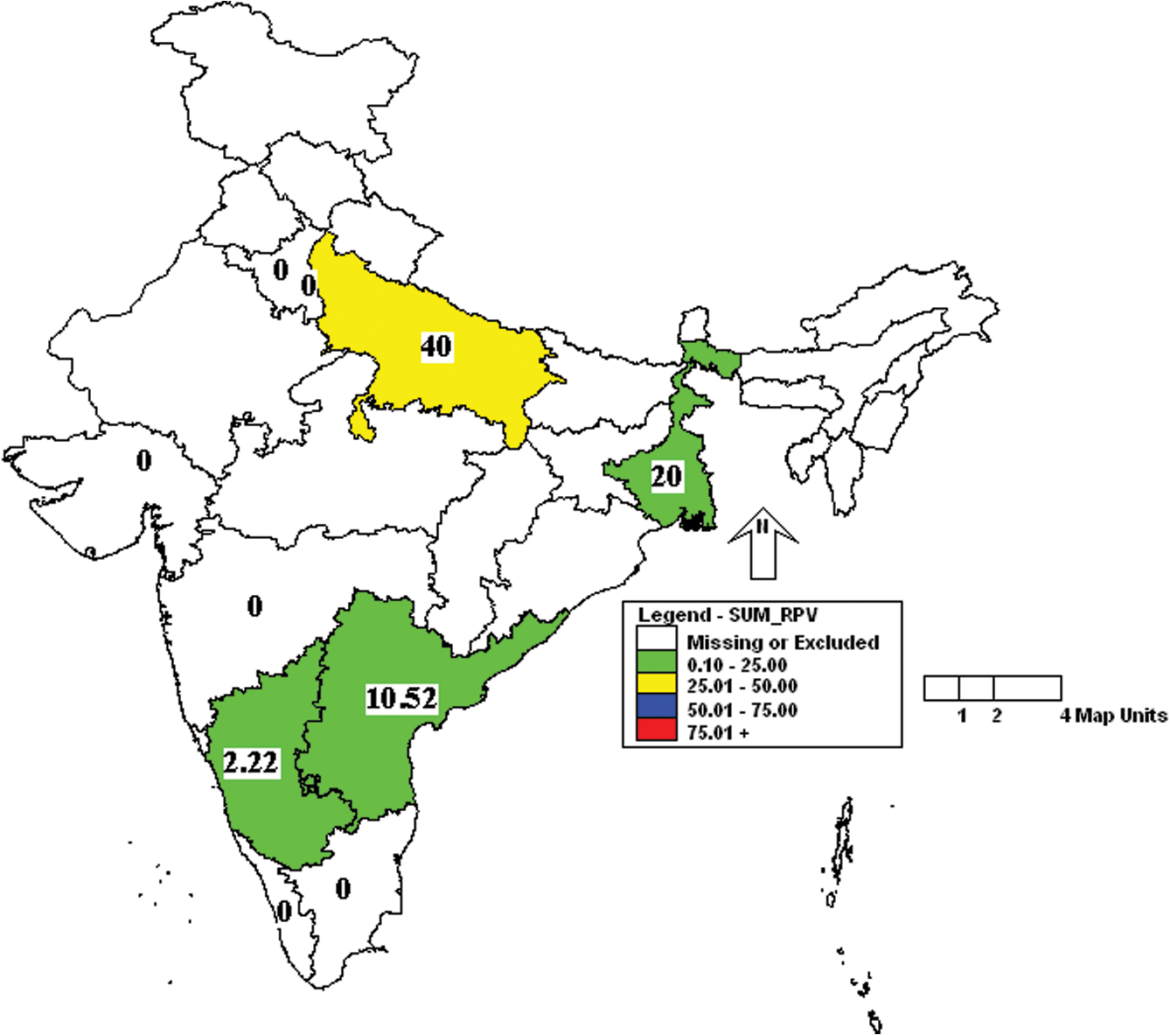
Incidence of rat parvovirus (RPV) in different states of India. Green—00.00 to 25.00%; Yellow—25.01 to 50.00%.

Further analysis of the results showed that the incidence of MHV was more in Uttar Pradesh and Kerala followed by Karnataka, West Bengal and Gujarat (Fig 1). Similarly, the incidence of *Mycoplasma pulmonis* was high in Uttar Pradesh, Delhi and Kerala (Fig 2). Prevalence of MVM was high in West Bengal compared to other states (Fig 3). However, SeV was detected only in Karnataka and West Bengal (Fig 4). Only one sample which was positive for LCMV was from West Bengal. The incidence of SDAV in rat colonies was observed in six different states whereas samples from four states were negative for the same (Fig 5). Samples from four states tested positive for RPV (Fig 6).

Data were also analyzed to determine the microbial load in public and private vivaria. In general, it was observed that the frequency of detection of pathogens was higher in vivaria of public institutes than animal facilities of private organizations. Particularly, the incidence of MHV and *Mycoplasma pulmonis* was 65.45% and 32.73% respectively, in public institutes but only 33.33% and 14.67% respectively, in private organizations. However, the incidence of MVM was more in private (17.33%) compared to public (9.09%) organizations (Table 4). On the other hand, there was not much difference in the incidence of rat pathogens between public and private vivaria (Table 4). Statistically, however, there was no difference overall in the incidence of rodent pathogens between public and private organizations (p = 0.9605). The results of various mouse (S1 Table) and rat (S2 Table) pathogens in various institutes are provided as supplementary information.

**Table 4:** Incidence of various pathogens in public (55 samples) and private (75 samples) vivaria.

Pathogen	Public	Private
Mouse hepatitis virus (MHV)	65.45%	33.33%
*Mycoplasma pulmonis*	32.73%	14.67%
Minute virus of mice (MVM)	9.09%	17.33%
Sendai virus (SeV)	0.00%	4.00%
Lymphocytic choriomeningitis virus (LCMV)	0.00%	1.33%
Ectromelia virus (ECTV)	0.00%	0.00%
Sialodacryoadenitis virus (SDAV)	12.73%	18.67%
Rat parvo virus (RPV)	7.27%	4.00%
Kilham’s rat virus (KRV)	0.00%	0.00%
Overall (no. of samples positive for at least one pathogen / total no. of samples)	53.33% for mice14.67% for rats	33.33% for mice25.33% for rats

## Discussion

The information on the prevalence rates of infections among laboratory animals has contributed to a better understanding of the epidemiology of these infections [16], as well as to judiciously implement health monitoring. The most common viral pathogens reported are norovirus, polyoma virus, K virus [17], [18], MHV, pneumonia virus of mice (PVM), reovirus, mouse parvovirus (MPV), rotavirus and SeV in mice, and PVM, SeV, SDAV, RPV, rotavirus and H-1 virus in rats [19]–[22]. Prevalence of various pathogens in mouse and rat colonies has been well documented in the US and Canada [18], [20], [23], Europe [16], [19], [21], Australia [24], South Africa [25], Brazil [26] and Argentina [27]. As far as Asia is concerned, reports are only available for Japan [28–29], South Korea [31], [32] and Taiwan [33]. There is no report documenting the prevalence of laboratory animal pathogens, except helminths, in India, and this is the first such report.

The prevalence rates of MHV, MVM and *M*. *pulmonis* in mice and SDAV and RPV in rats in India is much higher than that in developed countries where the incidence of any pathogen is typically less than 5% [7], [16], [24], [27], [30]. Of relevance to this study, prevalence rates of MHV, *M*. *pulmonis* and SeV in mouse colonies in South Korea and Taiwan, the closest Asian countries to India, range from 3.4% to 85%, 0.4% to 69% and 0% to 100%, respectively [31]–[33]. The prevalence rates of SDAV, RPV, KRV and SeV in rat colonies in South Korea and Taiwan range from 0% to 11.2%, 0% to 22.7%, 0% to 9.2% and 0% to 27.3%, respectively [31–33]. On the other hand, SeV and KRV prevalence in rats was lower in India than in Korea and Taiwan (0% versus up to 27.3% and 0% versus up to 6.25%, respectively). Our results show that although pathogen prevalence is mostly similar throughout the world, some inter-regional differences are apparent. We would like to point out that we did not test for the prevalence of murine noroviruses, which are reported to be highly prevalent among animal colonies worldwide.

Out of the six pathogens tested for mice, incidence of MHV was high in India. Being very susceptible to heat, detergents, desiccation and disinfectants, MHV would not be expected to sustain in the environment but its high prevalence may be due to its highly contagious nature [7], and the fact that it is shed in feces in large quantities for as long as four weeks after infection [34]. By contrast, the significant prevalence of parvoviral infections in rats can be attributed to their resistance to many of the common disinfectants, and stability in the environment [35] in addition to prolonged fecal shedding, leading to persistence of RPV in rat colonies [36]. On the other hand, the low incidence of MVM in mice could be due to the low sensitivity of these pathogens to serology-based tests, especially ELISA. Reportedly, MVM native antigens serve as poor targets over recombinant viral antigens for serological detection. In addition, similar disease can be caused by numerous genotypically different parvoviruses, producing false-positive reactions. In fact, recent studies in mice have shown that 90% of parvovirus infections detected by serology are due to MPV or related viruses rather than MVM, which appears to be responsible for only 5–10% of the parvovirus infections [22].

In comparison to corona and parvoviruses, ECTV and LCMV were rare or not recorded in the present study. Similar absence of serological evidence of LCMV and ECTV infection was noted in 1978 in the UK, and in the last decade in Australia [19], [24]. This could be due to some intrinsic characteristic/biology of these pathogens or that of the host or a combination of both.

The incidence of MHV and *M*. *pulmonis* was higher in laboratory animal colonies from public institutes compared to those of private organizations. A major contributor to this may be the use of barrier facilities by private organizations, which usually house a large number of transgenic animals. Despite the disagreement in prevalence, it must be noted that there was no overall statistically significant difference. However, further scrutiny revealed that the lack of overall significant difference could have been due to more number of public institutions each with fewer positive samples as against more number of positive samples in fewer private organizations, resulting in comparable proportions of positive samples overall. On the other hand, the incidence of rodent pathogens was significantly higher in North India compared to South India, and this may be due to the fact that more number of samples was from public entities. However, these results on either public versus private organizations or North versus South India should be interpreted with caution as the number of samples is low and not proportional to the colony size or to the density of vivaria in an area. Therefore the data have the potential to either overestimate or underestimate prevalence rates. Other limitations of the study include the small number of pathogens tested, as well as the lack of confirmatory tests. A more extensive study encompassing more organizations with more number of samples per organization over a period of time, which could not be done due to the prohibitive costs of the commercial kits, is needed to arrive at a more definitive conclusion.

## Conclusion

A preliminary study was carried out to determine the sero-epidemiology of rodent pathogens in public and private organizations in different parts of India. It was observed that MHV, *Mycoplasma pulmonis* and MVM were common in mouse colonies, and that SDAV and RPV were common in rat colonies. Presence of two or more pathogens was not uncommon especially in mice. By contrast, incidence was zero for ECTV in mice and SeV and KRV in rats. It was observed that incidence of MHV and *Mycoplasma pulmonis* were high in animal facilities, and the incidence of pathogens was significantly more in northern part of India.

## Supporting Information

S1 Table Results for various mice pathogens. Note: OD values >0.30 were considered as positive as per the instructions by the ELISA kit manufacturer.
(DOCX)

S2 Table Results for various rat pathogens. Note: OD values >0.30 were considered as positive as per the instructions by the ELISA kit manufacturer.
(DOCX)

## References

[1] Nicklas W, Homberger FR, Brunhilde IW, Jacobi K, Kraft V, Kunstyr I et al (1999) Implications of infectious agents on results of animal experiment. Lab Anim 33 (suppl.1):39–87.

[2] Collins MJ, Parker JC (1972) Murine virus contamination of leukemia viruses and transplantable tumors. J Natl Cancer I 49:1139–1143. 4343473

[3] Nicklas W, Kraft V, Meyer B (1993) Contamination of transplantable tumors, cell lines and monoclonal antibodies with rodent viruses. Lab Anim Sci 43: 296–300. 8231085

[4] Baker DG (1998) Natural pathogens of laboratory mice, rats and rabbits and their effects on research. Clin Microbiol Rev 11: 231–266. 9564563 10.1128/cmr.11.2.231 PMC106832

[5] Selwyn MR, Shek WR (1994) Sample sizes and frequency of testing for health monitoring in barrier rooms and isolators. Contemp Top Lab Anim 33: 56–60. 16466211

[6] Weisbroth SH, Peters R, Riley LK, Shek WR (1998) Microbiological assessment of laboratory rats and mice. ILAR J 39: 272–290. 11528088 10.1093/ilar.39.4.272

[7] Pritchett-Corning KR, Cosentino J, Clifford CB (2009) Contemporary prevalence of infectious agents in laboratory mice and rats. Lab Anim 43: 165–173. doi: 10.1258/la.2008.008009 19015179 10.1258/la.2008.008009

[8] Coates ME, Cooper JE, Heine W, Kraft V, Hedrich HJ (1994) Germ-free, gnotobiotic and specified pathogen-free (SPF) animals. In: Fujikura T et al., editor. Guidelines for the Breeding and Care of Laboratory Animals. Rome: World Health Organization (WHO) and International Council for Laboratory Animal Science (ICLAS), ISS/WHO/FAO-CC/IZSTe/94.23; pp 42–51.

[9] Festing MFW, Nomura T (1994) Animal quality control. In: Fujikura T, Govell GJR, Hänninen O, Pelkonen K., editor. Guidelines for the Breeding and Care of Laboratory Animals. Rome: World Health Organization (WHO) and International Council for Laboratory Animal Science (ICLAS), ISS/WHO/FAO-CC/IZSTe/94.23; pp 68–72.

[10] Mähler M, Berard M, Feinstein R, Gallagher A, Illgen-Wilcke B, Pritchett-Corning K et.al (2014) FELASA recommendations for the health monitoring of mouse, rat, hamster, guinea pig and rabbit colonies in breeding and experimental units. Lab Anim 48: 178–192. 24496575 10.1177/0023677213516312

[11] Loew FM, Fox JG (1983) Animal health surveillance and health delivery systems In: Foster HL, Small JD, Fox JG, editor. The Mouse in Biomedical Research, Vol 3 New York: Academic Press pp 69–82.

[12] Small JD (1984) Rodent and lagomorph health surveillance: Quality assurance In: Fox JG, Cohen BJ, Loew FM, editor. Lab Anim Med. New York: Academic Press pp 69–82.

[13] Nicklas W (1996) Health monitoring of experimental rodent colonies: An overview. Scand. J Lab Anim Sci 23: 69–75.

[14] Weisbroth S, Poe E (2000) Global harmonization of laboratory rodent health surveillance standards. Lab Anim 29: 43–47. 10.1038/5000067 11381226

[15] Voller A, Bartlett A, Bidwell DE (1978) Enzyme immunoassays with special reference to ELISA techniques. J Clin Pathol 31: 507–520. 78929 10.1136/jcp.31.6.507 PMC1145337

[16] Mahler M, Kohl W (2009) A serological survey to evaluate contemporary prevalence of viral agents and *Mycoplasma pulmonis* in laboratory mice and rats in Western Europe. Lab Anim 38: 161–165. 10.1038/laban0509-161 PMC7091905 19384313

[17] Poiley SM (1970) A survey of indigenous murine viruses in a variety of production and research animal facilities. Lab Anim Care 20: 643–650. 4318537

[18] Rowe WP, Hartley JW, Huebner RJ (1963) Polyoma and other indigenous mouse viruses. Lab Anim Care 13: 166–175. 14043135

[19] Carthew P, Verstraete A (1978) A serological survey of accredited breeding colonies in the United Kingdom for common rodent viruses. Lab Anim 12: 29–32. 415178 10.1258/002367778780953323

[20] Descoteaux JP, Grignon-Archambault D, Lussier L (1977) Serologic study of the prevalence of murine viruses in five Canadian mouse colonies. Lab Anim Sci 27: 621–626. 201796

[21] Gannon J, Carthew P (1980) Prevalence of indigenous viruses in laboratory animal colonies in the United Kingdom 1978–1979. Lab Anim 14: 309–311. 6257967 10.1258/002367780781071003

[22] Livingston RS, Riley LK (2003) Diagnostic testing of mouse and rat colonies for infectious agents. Lab Anim 32: 44–51. 10.1038/laban0503-44 PMC7091853 19757616

[23] Casebolt DB, Lindsey JR, Cassel GH (1988) Prevalence rates of infectious agents among commercial breeding populations of rats and mice. Lab Anim Sci 38: 327–329. 3411924

[24] McInnes EF, Rasmussen L, Fung P, Auld AM, Alvarez L, Lawrence DA et al (2011) Prevalence of viral, bacterial and parasitological diseases in rats and mice used in research environments in Australasia over a 5-y period. Lab Anim 40: 341–350. 10.1038/laban1111-341 PMC7091690 22012194

[25] Van Vuuren M, De Klerk WA, De Beer MC, VanNiekerk TA (1990) Survey for antibodies to selected viruses in laboratory mice in South Africa. J S Afr Vet Assoc 61: 174–175. 9022849

[26] Gilioli R, Sakurada JK, Andrade LA, Kraft V, Meyer B, Rangel HA (1996) Virus infection in rat and mouse colonies reared in Brazilian animal facilities. Lab Anim Sci 46: 582–584. 8905597

[27] Cagliada MP, Carbone C, Ayala MA, Laborde JM, Maschi F, Milocco SN et al (2010) Prevalence of antibodies against Kilham’s rat virus in experimental rat colonies of Argentina. Rev Argent Microbiol 42: 27–29. doi: 10.1590/S0325-75412010000100006 20461290 10.1590/S0325-75412010000100006

[28] Nakagawa M, Saito M, Suzuki E, Nakayama K, Matsubara J, Muto T. (1984) Ten-year long survey on pathogen status of mouse and rat breeding colonies. Exp Anim 33: 115–120. 10.1538/expanim1978.33.1_115 6088258

[29] Fujiwara K, Takenaka S, Shumiya S (1976) Carrier state of antibody and viruses in mouse breeding colony persistently infected with Sendai and mouse hepatitis viruses. Lab Anim Sci 26: 153–159. 178959

[30] Hayashimoto N, Morita H, Ishida T, Yasuda M, Kameda S, Uchida R et al (2013) Current microbiological status of laboratory mice and rats in experimental facilities in Japan. Exp Anim 62: 41–48. 23357945 10.1538/expanim.62.41

[31] Seok S, Park J, Cho S, Baek M, Lee H, Kim D et al (2005) Health surveillance of specific pathogen-free and conventionally-housed mice and rats in Korea. Exp Anim 54: 85–92. 15725684 10.1538/expanim.54.85

[32] Won YS, Jeong ES, Park HJ, Lee CH, Nam KH, Kim HC et al (2006) Microbiological contamination of laboratory mice and rats in Korea from 1999 to 2003. Exp Anim 55: 11–16. 16508207 10.1538/expanim.55.11

[33] Liang C-T, Shih A, Chang Y-H, Liu C-W, Lee Y-T, Hsieh W-C et al (2009) Microbial contaminations of laboratory mice and rats in Taiwan from 2004 to 2007. J Am Assoc Lab Anim Sci 48: 381–386. 19653946 PMC2715928

[34] Clifford CB, Watson J (2008) Old enemies, still with us after all these years. ILAR J 49: 292–302. 10.1093/ilar.49.3.291 PMC7108581 18506062

[35] Yang FC, Paturzo FX, Jacoby RO (1995) Environmental stability and transmission of rat virus. Lab Anim Sci 45: 140–144. 7603013

[36] Smith AL (1983) Response of weanling random-bred mice to inoculation with minute virus of mice. Lab Anim Sci 33: 37–39. 6339807

