# Supplementary material for: Sero-Prevalence of Rodent Pathogens in India
Source: PLoS One. 2015 Jul 9;10(7):e0131706. doi: 10.1371/journal.pone.0131706 (PMC4497729; doi:10.1371/journal.pone.0131706)
Supplement: S2 Table — Note: OD values >0.30 were considered as positive as per the instructions by the ELISA kit manufacturer. (DOCX) [file pone.0131706.s002.docx]

**S2 Table : Results for various rat pathogens**

| Institution | Sample # | KRV | SDA | RPV | Sendai |
| --- | --- | --- | --- | --- | --- |
| 1 | 1a | - | - | - | - |
|  | 1b | - | + | - | - |
|  | 1c | - | - | - | - |
|  | 1d | - | - | - | - |
|  | 1e | - | - | - | - |
| 2 | 2a | - | - | - | - |
|  | 2b | - | - | - | - |
|  | 2c | - | - | - | - |
|  | 2d | - | - | - | - |
|  | 2e | - | - | - | - |
| 3 | 3a | - | - | - | - |
|  | 3b | - | - | - | - |
|  | 3c | - | - | - | - |
|  | 3d | - | - | - | - |
|  | 3e | - | - | - | - |
| 4 | 4a | - | - | - | - |
|  | 4b | - | - | - | - |
|  | 4c | - | - | - | - |
|  | 4d | - | - | - | - |
|  | 4e | - | - | - | - |
| 5 | 5a | - | - | - | - |
|  | 5b | - | - | - | - |
|  | 5c | - | - | - | - |
|  | 5d | - | - | - | - |
|  | 5e | - | - | - | - |
| 6 | 6a | - | - | - | - |
|  | 6b | - | - | - | - |
|  | 6c | - | - | - | - |
|  | 6d | - | - | - | - |
|  | 6e | - | - | - | - |
| 7 | 7a | - | - | - | - |
|  | 7b | - | - | - | - |
|  | 7c | - | - | - | - |
|  | 7d | - | - | - | - |
|  | 7e | - | - | - | - |
| 8 | 8a | - | - | - | - |
|  | 8b | - | - | - | - |
|  | 8c | - | - | - | - |
|  | 8d | - | - | - | - |
|  | 8e | - | - | - | - |
| 9 | 9a | - | + | + | - |
|  | 9b | - | + | + | - |
|  | 9c | - | + | - | - |
|  | 9d | - | + | - | - |
|  | 9e | - | + | - | - |
| 10 | 10a | - | - | - | - |
|  | 10b | - | - | - | - |
|  | 10c | - | - | - | - |
|  | 10d | - | - | - | - |
|  | 10e | - | - | - | - |
| 11 | 11a | - | - | - | - |
|  | 11b | - | - | - | - |
|  | 11c | - | - | - | - |
|  | 11d | - | - | - | - |
|  | 11e | - | - | - | - |
| 12 | 12a | - | + | - | - |
|  | 12b | - | + | + |  |
|  | 12c | - | + | - | - |
|  | 12d | - | - | - | - |
|  | 12e | - | - | - | - |
| 13 | 13a | - | - | - | - |
|  | 13b | - | - | - | - |
|  | 13c | - | - | - | - |
|  | 13d | - | - | - | - |
|  | 13e | - | - | - | - |
| 14 | 14a | - | - | - | - |
|  | 14b | - | - | - | - |
|  | 14c | - | - | - | - |
|  | 14d | - | - | - | - |
|  | 14e | - | - | - | - |
| 15 | 15a | - | + | - | - |
|  | 15b | - | + | - | - |
|  | 15c | - | + | - | - |
|  | 15d | - | + | - | - |
|  | 15e | - | - | - | - |
| 16 | 16a | - | - | - | - |
|  | 16b | - | - | - | - |
|  | 16c | - | - | - | - |
|  | 16d | - | - | - | - |
|  | 16e |  |  |  |  |
| 17 | 17a | - | - | - | - |
|  | 17b | - | - | - | - |
|  | 17c | - | - | - | - |
|  | 17d | - | - | - | - |
|  | 17e | - | - | - | - |
| 18 | 18a | - | + | - | - |
|  | 18b | - | + | - | - |
|  | 18c | - | - | - | - |
|  | 18d | - | - | - | - |
|  | 18e | - | - | - | - |
| 19 | 19a | - | - | - | - |
|  | 19b | - | - | - | - |
|  | 19c | - | + | - | - |
|  | 19d | - | + | - | - |
|  | 19e | - | - | - | - |
| 20 | 20a | - | - | - | - |
|  | 20b | - | - | - | - |
|  | 20c | - | - | - | - |
|  | 20d | - | - | - | - |
|  | 20e | - | - | - | - |
| 21 | 21a | - | - | - | - |
|  | 21b | - | - | - | - |
|  | 21c | - | - | - | - |
|  | 21d | - | - | - | - |
|  | 21e | - | - | - | - |
| 22 | 22a | - | - | - | - |
|  | 22b | - | - | - | - |
|  | 22c | - | - | - | - |
|  | 22d | - | - | - | - |
|  | 22e | - | - | - | - |
| 23 | 23a | - | + | - | - |
|  | 23b | - | + | - | - |
|  | 23c | - | - | + | - |
|  | 23d | - | - | + | - |
|  | 23e | - | - | - | - |
| 24 | 24a | - | - | - | - |
|  | 24b | - | - | - | - |
|  | 24c | - | - | - | - |
|  | 24d | - | - | - | - |
|  | 24e | - | - | - | - |
| 25 | 25a | - | - | + | - |
|  | 25b | - | - | + | - |
|  | 25c | - | + | - | - |
|  | 25d | - | + | - | - |
|  | 25e | - | - | - | - |
| 26 | 26a | - | - | - | - |
|  | 26b | - | - | - | - |
|  | 26c | - | - | - | - |
|  | 26d | - | - | - | - |
|  | 26e | - | - | - | - |
| Total |  | 0 | 21 | 7 | 0 |

**Note : OD values >0.30 were considered as positive as per the instructions by the ELISA kit manufacturer.**
